# Extractable nitrogen and microbial community structure respond to grassland restoration regardless of historical context and soil composition

**DOI:** 10.1093/aobpla/plu085

**Published:** 2015-01-01

**Authors:** Sara Jo M. Dickens, Edith B. Allen, Louis S. Santiago, David Crowley

**Affiliations:** 1 Department of Botany and Plant Sciences, University of California Riverside, Riverside, CA 92521, USA; 2 Department of Environmental Sciences, University of California Riverside, Riverside, CA 92521, USA

**Keywords:** Carbon, exotic grasses, exotic plants, phospholipid fatty acid, resilience.

## Abstract

Grasslands have a long history of invasion by exotic annuals, which may alter microbial communities and nutrient cycling through changes in litter quality and biomass turnover rates. We compared plant community composition, soil chemical and microbial community composition, potential soil respiration and nitrogen (N) turnover rates between invaded and restored plots in inland and coastal grasslands. Restoration increased microbial biomass and fungal : bacterial (F : B) ratios, but sampling season had a greater influence on the F : B ratio than did restoration. Microbial community composition assessed by phospholipid fatty acid was altered by restoration, but also varied by season and by site. Total soil carbon (C) and N and potential soil respiration did not differ between treatments, but N mineralization decreased while extractable nitrate and nitrification and N immobilization rate increased in restored compared with unrestored sites. The differences in soil chemistry and microbial community composition between unrestored and restored sites indicate that these soils are responsive, and therefore not resistant to feedbacks caused by changes in vegetation type. The resilience, or recovery, of these soils is difficult to assess in the absence of uninvaded control grasslands. However, the rapid changes in microbial and N cycling characteristics following removal of invasives in both grassland sites suggest that the soils are resilient to invasion. The lack of change in total C and N pools may provide a buffer that promotes resilience of labile pools and microbial community structure.

## Abstract (precis)

Exotic plants have the capacity to establish positive plant-soil feedbacks facilitating invader persistence often at the cost of native plant species success. We compared the plant communities, soil chemical and microbial communities and nutrient turnover rates between invaded and restored plots in inland and coastal grasslands. Greater extractable N, slower N cycling rates and differing microbial community composition resulted from restoration. These differences indicate that grassland soils are responsive to vegetation change, and therefore not resistant to invasion-caused feedbacks. It also suggests these soils are resilient to invasion and may be capable of recovery following vegetative restoration.

## Introduction

The effects of exotic plant invasions on terrestrial ecosystems vary temporally and spatially and span scales ranging from the plant rhizosphere to changes in nutrient flux that occur at the ecosystem level (Ehrenfeld 2003; Potthoff *et al.* 2009). Previously, the impacts of exotic invasive plants on soil microbial communities and nutrient fluxes have received considerable attention (e.g. Jackson *et al.* 1988, 1989; Bever *et al.* 1997; Hawkes *et al.* 2005, 2006; Wolfe and Klironomos 2005). However, belowground responses to restoration practices and studies on the legacy effects of plant invasions are relatively new areas of research (Potthoff *et al.* 2006, 2009; Kulmatiski and Beard 2008, 2011; Dickens 2010; Dickens and Allen 2014). The capacity of invaded systems to recover from short-term and legacy effects of exotic plants is unknown. In addition, the role of legacy effects of exotic invasion and exotic species identity in the success of restoration is unclear, greatly limiting the knowledge base needed for strategic restoration of invaded lands.

One mechanism by which exotic plants impact ecosystems is by decoupling plant–soil feedback loops that previously functioned in soils under native vegetation. We define decoupling here and the interruption of interactions between plants and soil via soil inputs and microbial community responses (Bardgett *et al.* 2013). Feedback loops describe how plants, soils and microorganisms interact through resources. For example, a plant species may produce particular soil inputs via senescent biomass and exudates that become resources for soil microbes. Microbes that use these resources determine rates of nutrient cycling and thus nutrient availability to plants. Through this feedback loop, plants and microbes may exert selective pressure on one another (Wardle 2002; Eviner and Chapin 2003; Santiago *et al.* 2005; Santiago 2007). In the case of plant invasion, a new species' arrival may alter the microbial community, leading to further modifications of belowground processes such as nutrient turnover or the introduction of microbial species associated with this novel plant. The end result can be inhibition of native plant species and/or the facilitation of the invading, exotic plant species (Bever *et al.* 1997; Ehrenfeld 2003; Wolfe and Klironomos 2005).

Introductions of plant species that differ in litter quality, phenology and relative distribution of above and belowground biomass may result in especially strong plant–soil feedbacks. Exotic species may introduce novel nutrient uptake or litter deposition traits that could create positive feedbacks with the soil microbial community (Grayston *et al.* 1998; Eviner 2004; Batten *et al.* 2006). Exotics may shift the seasonal availability of extractable nitrogen (N) by introducing phenologies with earlier germination and growth rates (Jackson *et al.* 1988; Dickens 2010; Dickens and Allen 2014) and changes in soil properties that drive the selection and composition of microbial communities (Ehrenfeld 2003; Wardle *et al.* 2004; Berg and Smalla 2009; Potthoff *et al.* 2009). Additionally, exotic plant invasion can change cycling and availability of C, N and other nutrients (Christian and Wilson 1999; Ehrenfeld 2003; Yoshida and Allen 2004). Litter with high C : N promotes immobilization of N by microbes resulting in reduced available N (Brady and Weil 1996; Grayston *et al.* 1998; Cione *et al.* 2002; Potthoff *et al.* 2009). Invasion of exotic, annual grasses into a perennial bunchgrass grassland would be expected to introduce litter of lower C : N compared with native perennials which would increase decomposition and N cycling rates (Eviner and Firestone 2007; Potthoff *et al.* 2009).

California grasslands are highly invaded by exotic annuals and undergoing restoration in many locations, and thus an ideal system for studying plant–soil feedbacks through decoupling exotic plant species' plant–soil feedbacks using restoration. Plant biomass in grasslands turns over annually (Jackson *et al.* 1988) so grassland soils are likely to respond to altered plant inputs over a relatively short time scale. Due to the almost complete conversion of native perennial grasslands with annual forbs to exotic annual grassland with annual forbs (Biswell 1956; D'Antonio 2007; Minnich 2008), native California grasslands are a system of high conservation value and concern. Annual plant invasions began >200 years ago (Minnich 2008), and invasion is so widespread that there are no true relic grasslands to use as reference sites. However, even without relic grasslands, differences in soil microbial community structure, soil chemistry and nutrient flux rates between unrestored and restored soils can be used to evaluate the capacity of grassland soils to respond to changes in vegetation type.

**Figure 1.**
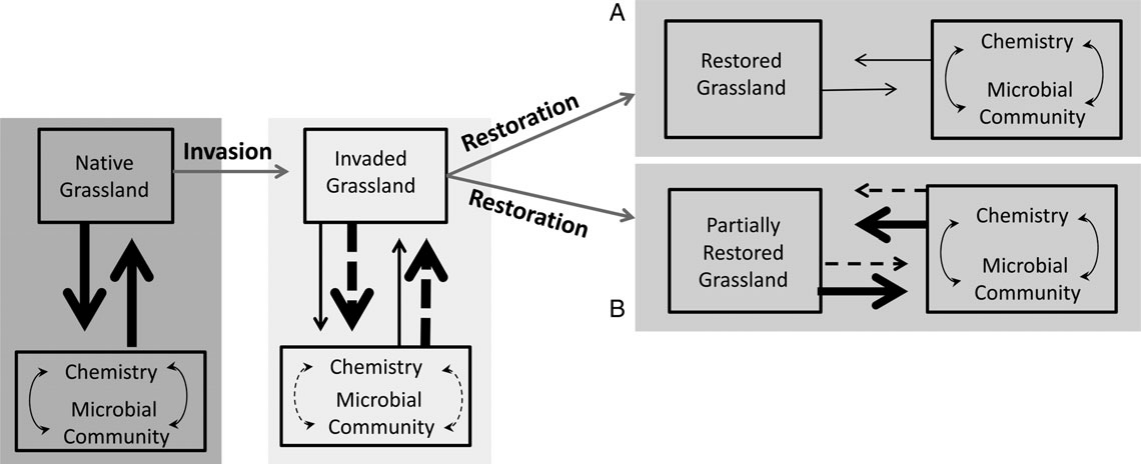
Invasion of original, native grasslands introduced new, exotic plant–soil feedbacks (solid black arrows represent native feedbacks and dotted black arrows represent plant soil feedbacks of exotics). Through restoration efforts plant–soil feedbacks can be altered leading to (A) a restored grassland experiencing no exotic feedbacks and moderate native feedbacks from the re-establishing native community or (B) a partially restored grassland experiencing a much higher proportion of native plant–soil feedbacks than exotic. Thickness of arrows indicates the degree to which feedbacks are influencing the system.

Few studies have observed soil recovery after removal of invasives and native species restoration (but see Potthoff *et al.* 2006; Kulmatiski and Beard 2008, 2011; Dickens and Allen 2014). Shifts in microbial community structures can occur within a few years of plant species community compositional changes and microbial abundances may remain affected by land-use legacies for 50 years (Kulmatiski and Beard 2008). Further studies are necessary to determine which system responses are capable of rapid recovery or slower re-establishment of native feedback loops and whether patterns of responses are similar across differing environments. The objective of this study was to assess the capacity of southern California grassland soils to diverge from their invaded condition following the decoupling of long-term exotic plant–soil feedbacks. Invasion has likely led to the establishment of exotic plant–soil feedbacks that overwhelm feedbacks produced by the limited native plant population. Through restoration there are two possible, successful restoration scenarios. The first is successful removal of exotics and their associated plant–soil feedbacks leaving the restored grassland with limited native cover and bare ground initially. The second is a partially restored grassland that is dominated by native plant–soil feedbacks but still experiences some exotic plant–soil feedbacks due to constant, but limited, reinvasion (Fig. 1). We hypothesized that (i) restoration by removing exotic annual grasses will lead to shifts in the microbial community, reflecting the development of new plant–soil feedbacks; (ii) exotic annuals often have higher quality litter than native perennial grasses, so microbial community shifts would translate into reduced carbon (C) and N cycling rates following restoration; and (iii) soil responses to restoration are sensitive to environmental conditions, which will result in different magnitudes of shifts in both microbial community and nutrient cycling at sites with different land-use/management histories and environmental contexts.

## Methods

We investigated inland and coastal grassland sites in southern California that have been invaded by Mediterranean annual grasses and forbs, but that still support sparse native bunchgrasses and forbs. No uninvaded reference grasslands occur in southern California (Minnich 2008). An important contextual factor in this study is the difference in site histories, soils and current management strategies applied between our two study sites (Table 1). The inland grassland is located within the 4000-ha Santa Rosa Plateau Ecological Reserve in Murrieta, CA (33°31′N, 117°15′E). Soils at this location are basalts of the Vallecitos loam, thick solum variant (USDA NCSS SoilWeb Network), and restoration consisted of exotic grass control through prescribed spring burns but no reseeding (Gillespie and Allen 2004). The 120-ha White Point Preserve coastal grassland is located in San Pedro, Los Angeles County (33°43′N, 118°18′W), and soils are classified as a clay loam of the Diablo Clay Adobe series (Nelson *et al.* 1919). Restoration consisted of hand weeding and mowing of invasive plant species and reseeding of native species. To examine the effects of restoration on the structure and function of the invaded grassland, we used nine previously established 1 m^2^ plots within areas that had undergone long-term restorations (9 years) and an additional nine plots in an adjacent unrestored grassland at each location. Restored areas were defined as those having experienced active restoration that had an exotic plant species cover of <40 % and were dominated by native species, while unrestored areas had ≥50 % cover by exotic plant species with sparse native species.

**Table 1.** Comparison of site abiotic properties, land-use history and restoration methodologies.

	Inland	Coastal
Mean annual precipitation (cm)	48	30
Annual temperature range (°C)	1–37	8–26
Soil clay (%)	12	36
Soil silt (%)	57	35
Soil sand (%)	31	29
Elevation (m)	579	47
Land-use history	Grazing	Defence missile facility
Year of restoration	1997	2000
Restoration method	Prescribed burn	Mowing, hand seeding, irrigation

### Measurements of ecosystem structure

Plant species richness, per cent cover by individual species and per cent of litter cover were measured annually by visual estimation in gridded 1 m^2^ frames in each treatment at the peak of the growing season (March) in 2007–09. Net annual productivity of annuals was determined by harvesting biomass in four functional groups (native forb, native grass, exotic forb and exotic grass) clipped at the soil level from 0.25 m^2^ sub-plots and scaled up to the 1 m^2^ plot size using regression of plant biomass and per cent cover in 0.25 m^2^ and per cent cover of 1 m^2^ plots. Additional biomass was collected for chemical analysis of the vegetative plant tissue at peak plant growth. All biomass was oven dried at 60 °C and weighed. Biomass for tissue analysis was ground and analysed for total C and N on a soil combustion analyser system (Flash AlllZ, Thermo-Finnigan).

To determine the effects of restoration on soil biological and chemical characteristics, three soil cores of 2.5 cm diameter and 10 cm depth were collected per plot, composited to the plot level and then transported on ice to the laboratory where a portion of each sample was stored at −20 °C until processed for chemical analyses and the remaining portion of the sample at −80 °C for microbial analyses. Soils were analysed for total C and N by combustion, KCl-extractable NO_3_ and NH_4_, and bicarbonate-extractable phosphorus (Olsen P) by the University of California Analytical Laboratory at UC Davis (anlab.ucdavis.edu). Soil pH was measured using a 2 : 1 soil : water slurry. Soil cores were collected once annually in 2007 and 2009 at peak growth, and three times annually (at germination, peak plant growth and plant senescence) during 2007–08 for analysis of KCl-extractable N ($\text{N}{{\text{H}}_{4}}^{+}$ and $\text{N}{{\text{O}}_{3}}^{-}$).

Phospholipid fatty acid (PLFA) analysis was used to determine whether microbial community structure was affected by restoration of the native vegetation. With the exception of Archaea, all other living organisms contain PLFAs as a component of their cellular membranes (White *et al.* 1996; Hedrick *et al.* 2000). These compounds can be used as biomarkers to identify functional groups of microbes such as Gram-positive bacteria or arbuscular mycorrhizal (AM) fungi (Zelles and Bai 1994; White *et al.* 1996; Hedrick *et al.* 2000). Phospholipid fatty acids are preferable to the use of fatty acids alone as fatty acids can persist in soils for long periods of time representing a legacy of past microbial communities. Phospholipid fatty acid represent living organisms (White *et al.* 1996), thus ensuring capture of the current microbial community response to a disturbance such as exotic plant invasion or restoration activities. Samples were collected within 24 h of rainfall or wetting of soils to a 10-year average rainfall volume. Soil samples were passed through a 2-mm sieve and lyophilized prior to extraction. Phospholipid fatty acids were extracted from 6 g of soil following the modified Bligh–Dyer method (Frostegard *et al.* 1991). Quantification of fatty acids was obtained using a gas chromatograph (HP6980; Hewlett Packard, Palo Alto, CA, USA) with a flame ionization detector and HP3365 ChemStation Software. Phospholipid fatty acid peaks were converted to PLFA identities and abundances using MIDI Sherlock Microbial Identification System (MIDI, Inc., Newark, NJ, USA) followed by comparison of peak areas with a known internal standard 19 : 0 of known concentration. Bacterial biomarkers included: 14 : 0, 15 : 0 iso, 15 : 0 antiso, 16 : 0 iso, 16 : 0 iso G, 16 : 1 w9c, 16 : 1 w7c, 16 : 0, 16 : 1 2OH, 17 : 1 alcohol, 17 : 0 iso, 17 : 0 antiso, 17 : 0 cyclo, 17 : 1 w8c, 18 : 1 w5c, 18 : 0, 19 : 0 cyclo c11–12, 22 : 0 and 24 : 0 and fungi: 18 : 2 w6c, 18 1w9c and 17 : 0 and AM fungi: 16 : 1 w5c. Nomenclature for PLFAs followed Lechevalier and Lechevalier (1988), Vestal and White (1989), Zelles (1999), Myers *et al.* (2001) and Hebel *et al.* (2009).

### Measurements of ecosystem function

Laboratory incubations for potential N mineralization were performed over a 30-day period in soil samples maintained at 25 °C and 60 % humidity. $\text{N}{{\text{H}}_{4}}^{+}\text{and}\;\text{N}{{\text{O}}_{3}}^{-}$ were extracted with a 2-M KCl 4 : 1 solution (Riley and Vitousek 1995) and shipped on dry ice for analysis at the University of California Analytical Laboratory at UC Davis (anlab.ucdavis.edu). Net mineralization was calculated as the change in $\text{N}{{\text{H}}_{4}}^{+}$ minus the change in $\text{N}{{\text{O}}_{3}}^{-}$ over time, and net nitrification was calculated as the change of $\text{N}{{\text{O}}_{3}}^{-}$ over time following Riley and Vitousek (1995). Potential soil respiration rates were determined using laboratory incubations. Soils were maintained at 20 % soil moisture and 25 °C in sealed glass jars for 10 days. Jar headspace concentrations of CO_2_ (ppm) were determined using a LiCor 800 infrared gas analyser (Lincoln, NE, USA) and converted to a rate function of μmol CO_2_-C/g soil × day (Chatterjee *et al.* 2008).

Plant species per cent cover and richness were analysed using repeated-measures multivariate analysis of variance (MANOVA) to assess how the vegetative community responded to restoration. Plant biomass and litter, soil chemistry, soil-extractable N, potential soil respiration and N mineralization data were analysed with ANOVA followed by Tukey's HSD to determine whether restoration altered soil chemical pools and cycling rates. Non-normal data were log(*x*+ 1) or square root transformed when appropriate and a Kruskal-Wallis nonparametric test was performed in cases where the data could not be transformed to normality. Microbial biomass and F : B were analysed using ANOVA to determine coarse microbial community compositional shifts between treatments. Principal component analysis (PCA) was used to create ordination diagrams to compare microbial community compositions, which were then further analysed by ANOVA of PC1 and PC2 values to determine if community composition differed following restoration and across sampling dates. The analyses were conducted using JMP9 (SAS Institute 2009) with an alpha level of *P* ≤ 0.05.

## Results

Restoration shifted plant species dominance from exotic to native grassland plant species. More specifically, restoration reduced exotic forbs by 59 % at the inland site and 75 % at the coastal site and exotic grasses by 15 % at the inland and 39 % at the coastal site. There was also a 79 and 93 % increase in native grasses at inland and coastal sites, respectively (Table 2). A complete species list and individual cover values are reported in Dickens (2010). Restoration promoted a shift in the quality and quantity of aboveground litter inputs to soil. Litter cover was 25 % higher in restored plots (*P* < 0.0001) than in unrestored plots at the inland site during germination and 38 % higher at senescence (*P* = 0.0002) but litter cover was unaffected by restoration at the coastal site. In 2007, the drought year, the coastal site accumulated 30 % greater litter than the inland site, but in 2008, an average rain year, 85 % less litter cover than the inland site (*P* < 0.0001 both years). Restoration at the inland grassland site led to a 300 % increase in native grass biomass (*P* < 0.001). Biomass data for the coastal site was not available because plots were unintentionally destroyed during management practices prior to biomass collection. Plant tissue C content varied across all species tested (Table 3). *Erodium brachycarpum (*decreased by 50 % inland), *Brassica nigra* (decreased by 112 % coastal) and *Avena barbata* (increased by 48 % inland and 100 % coastal) had the lowest leaf tissue N concentrations, whereas the exotic grasses *Brachypodium distachyon* (decreased by 54 % coastal) and *Bromus rubens* (decreased from 3 to 0 %) had the highest. The native grass, *Stipa pulchra,* had an intermediate N concentration and increased by 39 % (inland) and 40 % (coastal) (Table 3). Overall changes in tissue chemistry appear small, but in fact species with the most different tissue chemistry from the native *S. pulchra* are the species that decreased the most with restoration leaving those more similar to *Stipa* as dominant exotic species.

**Table 2.** Common species mean per cent cover of inland and coastal grassland plant functional groups during the peak of the 2007–08 season. Repeated-measures MANOVA were conducted to assess differences in plant composition between treatments of unrestored and restored grasslands over 3 years during the 2006–09 growing seasons.

Grassland type	Functional groups	Unrestored	Restored	*P*-values		
				Treatment	Time	Time × treatment
Inland grassland	Native grass	8.3 (1)	40.1 (1)	<0.0001	<0.0001	0.071
	Native forb	4.1 (10)	3.2 (9)	0.015	<0.0001	0.047
	Exotic forb	59.6 (8)	24.7 (5)	0.073	<0.0001	<0.0001
	Exotic grass	47.0 (5)	39.7 (4)	0.372	<0.0001	0.009
Coastal grassland	Native shrubs	2.4 (1)	0.0 (0)	0.362	0.670	0.670
	Native grasses	3.0 (1)	41.5 (1)	<0.001	0.074	0.048
	Exotic forbs	50.8 (5)	12.9 (4)	<0.001	<0.001	0.001
	Exotic grasses	67.8 (2)	41.5 (2)	0.066	<0.001	0.196

**Table 3.** Plant leaf tissue chemical composition for some of the most common species encountered at the two project sites. Five samples of each plant species were analysed and averaged per species.

Functional group	Species	N	C	C/N
Native grass	*S. pulchra*	1	42.7	42.1
Exotic grass	*A. barbata*	0.7	41.2	62
	*B. distachyon*	1.3	42.3	31.3
	*B. rubens*	1.7	42.5	25.5
	*Festuca myuros*	0.9	42.6	49.7
Exotic forb	*E. brachycarpum*	0.6	42.7	67.4
	*B. nigra*	0.9	41.2	56.5

**Figure 2.**
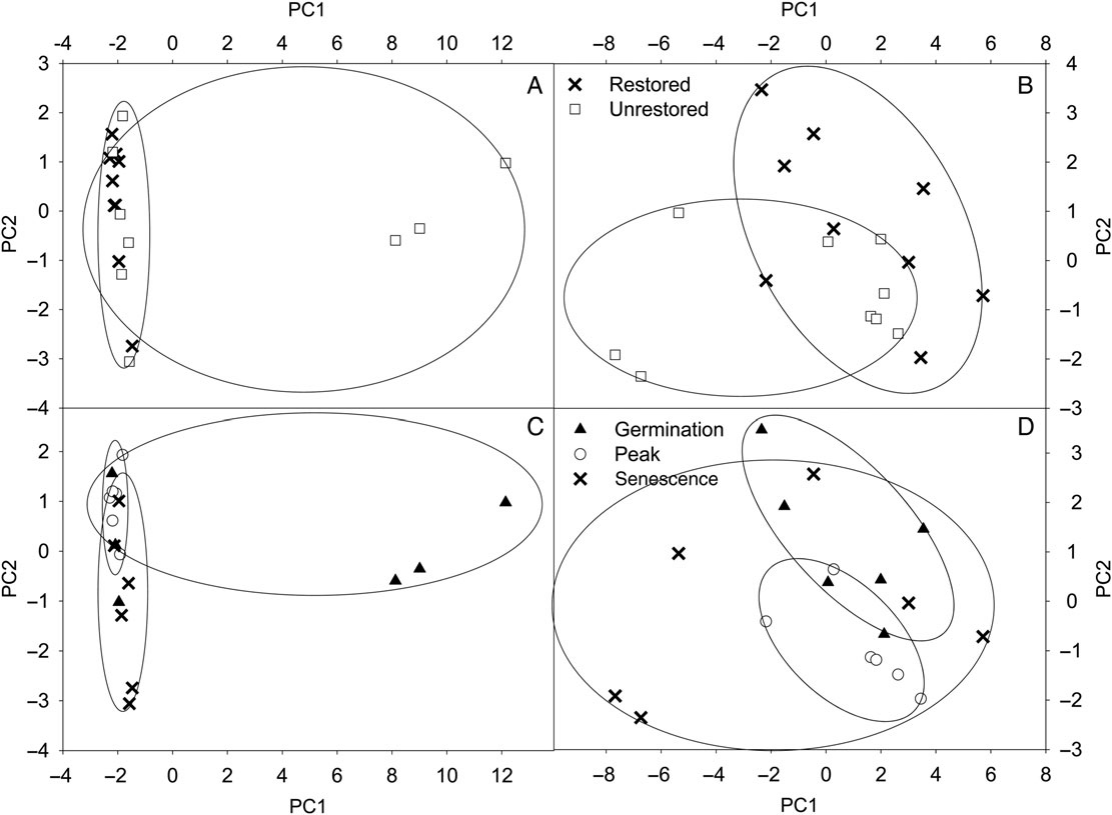
Principal component analysis results for PLFA microbial community analysis at the inland site (A and C) and the coastal site (B and D) during the 2007–08 growing season. Restored data points of graph (A) refer to restored-burned treatments of the inland site and those of graph (B) refer to restored-weeded treatments of the coastal site. Graphs (A) and (B) assess differences between treatment while (C) and (D) assess differences between sampling dates. PC1 explains 83 % variation and PC2 has a cumulative per cent variation of 91 % for the inland site, while the coastal site cumulative variance explained by PC1 is 59 % and PC2 is 70 %. Ellipses indicate statistically different microbial communities determined by ANOVA of PC values.

Restoration led to shifts in microbial biomass, microbial community structure and fungal : bacterial (F : B) ratio, but shifts were variable across seasons. Microbial biomass was 29 times lower following restoration at the inland site during germination (Table 4). However, microbial biomass was approximately doubled with restoration during senescence at the coastal site. Fungal : bacterial ratios, while not different between unrestored and restored treatments at the inland site, increased at the coastal site restored plots during season peak, but were lower during plant senescence (Table 4). The greatest numbers of PLFA biomarkers at both sites were from bacterial functional groups with markers for fungi, protozoa and proteobacteria in lower abundance (Table 4). The inland site also had biomarkers for microeukaryotes and pseudomonads in low abundances. Concentrations of biomarkers from all functional groups except microeukaryotes and *Pseudomonas* differed between unrestored and restored plots during plant germination. Soils sampled during plant senescence at the coastal site and at germination at the inland site had increased AM fungal marker 16 : 1 w5c **[****see Supporting Information****]**. The microbial community as a whole, as defined by PLFA biomarkers was differentiated by both restoration treatment and season at both sites (Fig. 2; **see Supporting Information**). There were several between-site differences in response to restoration microbial communities differed in both functional group and mass between sites and within the growing season. The inland site generally had greater microbial biomass and AM fungi than the coastal site, but sites had similar F : B ratios except at senescence when coastal F : B ratios nearly doubled. There was a similar decrease in average microbial biomass between the two sites over the season (inland = 16 %, coastal = 13 %).

**Table 4.** The common PLFA biomarkers (μmol PLFA g^−1^ soil) and corresponding microbial taxa from the inland and coastal grasslands and between sites during the 2007–08 season. Means are shown for biomarkers making up >2 % of total PLFA abundance. Asterisks indicate the level of significance between treatments. *P ≤ 0.1, **P ≤ 0.05 and ***P ≤ 0.001 determined with ANOVA.

Grassland type	Microbial functional group	Germination		Peak		Senescence	
		Unrestored	Restored	Unrestored	Restored	Unrestored	Restored
Inland grassland	General	240 048	53 142***	21 534	28 807	34 587	33 510
	General bacteria	1 470 719	124 444***	59 108	125 379	183 234	152 469
	Gram positive	897 933	77 569**	76 223	83 427	107 136	91 697
	Gram negative	414 437	34 637***	35 871	38 600	58 637	46 054
	Fungi	695 573	46 975*	63 432	55 442	82 956	72 111
	AM fungi	135 076	11 818***	12 502	15 280	19 198	16 899
	Microeukaryote	21 184	2086	0	2073	3065	3073
	Protozoa	5 216 391	0**	1188	1131	1112	1048
	Proteobacteria	0	2800***	2074	2019	3364	3314
	*Pseudomonas*	13 969	2187	1756	1495	3164	2396
	Microbial biomass	7 792 960	267 741**	252 280	267 362	362 341	314 489
	F : B	0.461	0.382	0.555	0.414	0.461	0.469
Coastal grassland	General	14 022	10 959	18 573	15 032	6007	16 676**
	General bacteria	3386	3422	4226	3940	708	4313**
	Gram positive	24 697	18 450	25 853	21 221	12 092	24 348*
	Gram negative	15 204	13 713	14 417	12 504	2082	15 775**
	Fungi	15 408	16 942	16 988	17 943	13 269	21 107*
	AM fungi	4893	3539	5093	4034*	850	5245**
	Protozoa	665	2346	691	293	0	505
	Proteobacteria	9574	9975	10 280	10 059	9771	14 262*
	Microbial biomass	90 565	82 187	97 554	86 315	45 193	105 756**
	F : B	0.362	0.465	0.382	0.476***	0.975	0.481**
Between site	Inland	Coastal	Inland	Coastal	Inland	Coastal	
	General	146 595	12 491**	25 171	16 802	34 049	11 341***
	General bacteria	797 581	3404**	130 267	4083***	167 852	2510***
	Gram positive	487 751	21 573**	79 825	23 537***	99 417	18 220***
	Gram negative	224 537	14 458**	37 236	13 461***	52 345	8929***
	Fungi	371 274	16 175**	59 437	17 466***	77 534	17 188***
	AM fungi	73 447	4216**	13 891	4563***	18 049	3047***
	Microeukaryote	11 635	0*	1037	0	3069	0***
	Protozoa	2 608 196	1506*	1160	492	1081	253
	Proteobacteria	1400	9775***	2047	10 169***	3339	12 016***
	*Pseudomonas*	8078	0*	1696	0**	2780	0***
	Microbial biomass	4 030 350	86 376*	259 821	91 935***	338 415	75 475***
	F : B	0.421	0.413	0.485	0.429	0.465	0.728*

**Figure 3.**
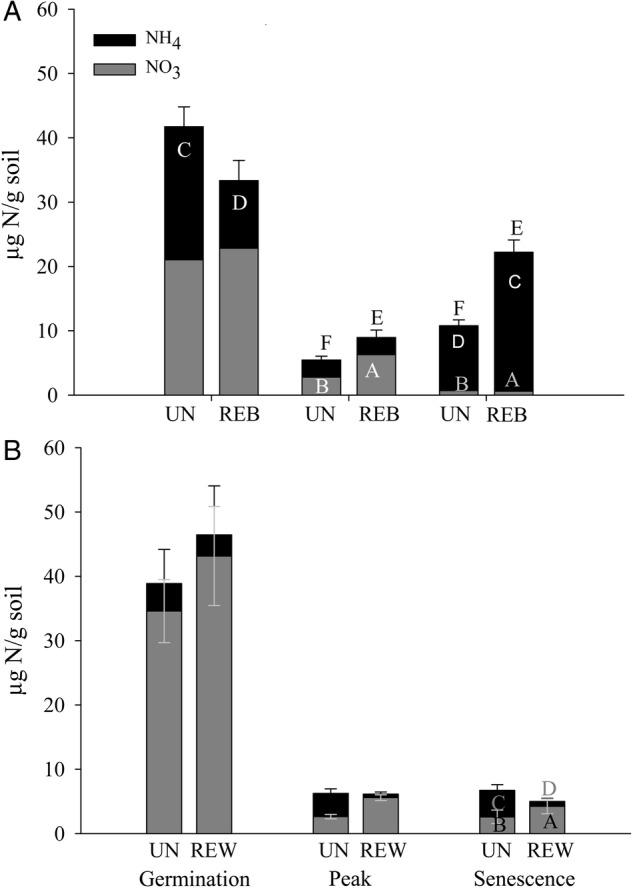
Soil-extractable N during the 2007–08 season at the inland site (A) and the coastal site (B). Treatments are: UN = unrestored, REB = restored by burning at the inland site and REW = restored by weeding and mowing at the coastal site. Letters indicate significant differences using ANOVA followed by Tukey–Kramer HSD test: NO_3_ = A and B, NH_4_ = C and D and total extractable N = E and F. Bars indicate standard error and letters significant differences (*P* ≤ 0.05).

Chemical properties of soil N shifted in the form of both increased and reduced NH_4_-N according to the season and site, increased NO_3_-N and total extractable N and altered N cycling rates. Restoration of the inland site reduced NH_4_-N during germination in the 2007–08 season (*P* = 0.004), but increased NH_4_-N during the peak of the growing season (*P* = 0.009; Fig. 3). In contrast, restoration of the coastal site only reduced NH_4_-N during senescence (*P* = 0.020; Fig. 3B). Restoration leads to a greater availability of NO_3_-N (*P* = 0.058) and total extractable N (*P* = 0.0002) during the peak season at the inland site and of NO_3_-N during senescence at both sites (inland *P* = 0.005; coastal *P* = 0.005; Fig. 3A and B). Restoration did not impact total extractable N at the coastal site. Peak season extractable N patterns were consistent across the 3 years of 2007–09 at the inland site where total extractable N (2007, *P* = 0.040, 2008, *P* = 0.002, 2009, *P* = 0.001) and NO_3_-N in 2007 (*P* = 0.041) and 2009 (*P* < 0.001) increased following restoration. The coastal site had increased total extractable N but reduced NO_3_-N with restoration in the drought year, 2007, with no differences in any form of extractable N in 2008.

**Figure 4.**
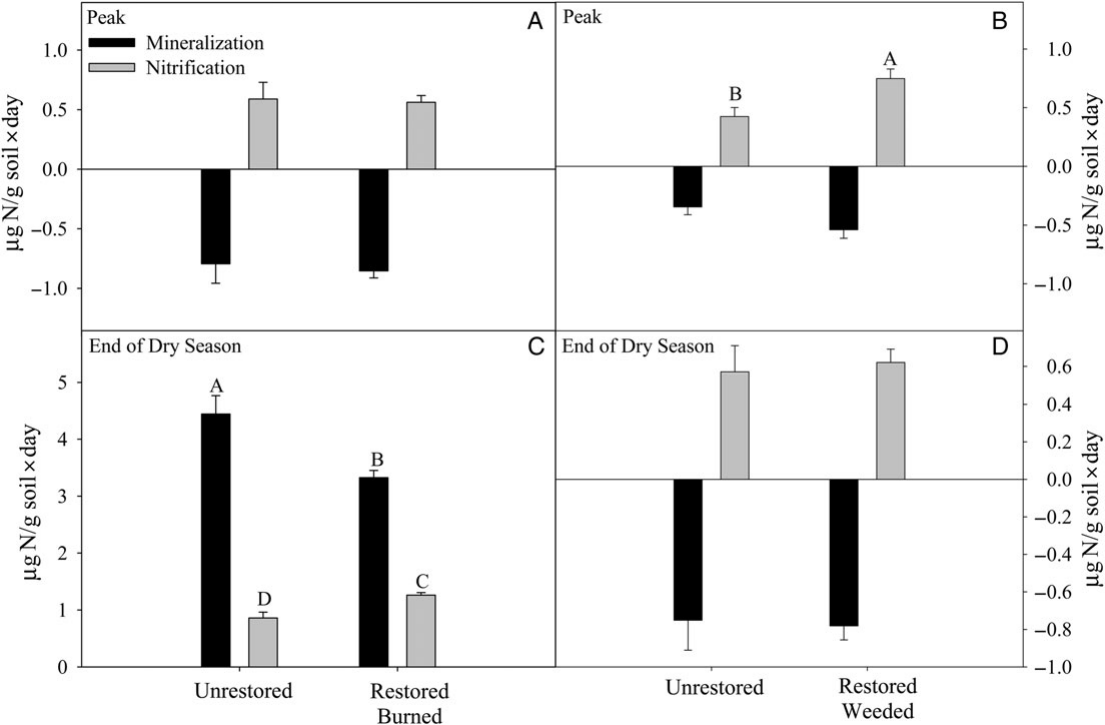
Potential N mineralization and nitrification from 30-day laboratory incubations from the inland site (A and C) and the coastal site (B and D) for soils collected in the March (peak) and August (end of the summer dry season) of 2008. Letters indicate significant differences using the Tukey–Kramer HSD test following ANOVA (*P* ≤ 0.05). Bars indicate standard error.

Total soil N and C, pH and P were unaffected by restoration (Table 5). Soil potential respiration was unaffected by restoration at the inland grassland sites. Plots at the coastal site were unintentionally destroyed before soil respiration sampling was conducted so potential soil respiration data were not available for that site. Potential N mineralization was reduced by restoration only in August soils and only at the inland site (*P* = 0.017). Potential nitrification rates were increased with restoration for soils collected in August at the inland site (*P* = 0.002; Fig. 4C) and in March at the coastal site (*P* = 0.011; Fig. 4B).

**Table 5.** Soil chemical data (means and standard errors) for the burned, inland site and the coastal site collected the summer of 2006. ANOVA was conducted to assess differences in soil chemical characteristics between treatments of unrestored and restored grasslands during the 2006–07 growing season.

	Inland unrestored	SE	Inland restored-burned	SE	Inland treatment *P*-value	Coastal unrestored	SE	Coastal restored-weeded	SE	Coastal treatment *P*-value	Inland site	SE	Coastal site	SE	Site *P*-value
Total N (%)	0.2	0.01	0.2	0.00	1.000	0.2	<0.10	0.2	<0.10	0.162	0.2	0.0	0.2	0.0	<0.0001
Total C (%)	2.2	0.08	2.2	0.06	0.885	3.2	0.20	3.1	0.20	0.727	2.2	0.1	3.2	0.1	<0.0001
Soil organic matter (%)	8.5	0.30	8.0	0.20	0.136	13.3	0.30	13.0	0.20	0.541	8.3	0.2	13.1	0.2	<0.0001
C/N	13.4	0.07	13.3	0.14	0.499	14.0	0.53	15.7	0.80	0.105	13.4	0.1	14.9	0.5	0.007
NH_4_ (µg g^−1^)	6.5	0.25	9.2	2.43	0.284	7.4	0.28	8.3	0.60	0.308	7.8	1.2	7.8	0.4	0.997
NO_3_ (µg g^−1^)	3.6	1.66	1.8	0.45	0.295	5.8	1.31	31.9	8.30	0.001	2.7	0.9	18.8	5.2	0.004
Total extractable N (µg g^−1^)	10.1	1.84	11.0	2.38	0.783	13.1	1.51	40.2	8.60	0.007	10.5	1.5	26.7	5.4	0.006
Olsen-P (µg g^−1^)	5.1	0.71	4.1	0.79	0.351	18.8	1.10	22.1	3.60	0.401	4.6	0.5	20.4	1.9	<0.0001
pH	5.9	0.07	5.9	0.04	0.980	8.0	<0.10	8.0	0.10	0.537	5.9	0.0	8.0	0.0	<0.0001

## Discussion

Microbial shifts in community structure and altered extractable N pools and N cycling indicate that exotic plant–soil feedbacks were decoupled following restoration at both sites regardless of differences in soil properties between sites. Decoupling is used here to mean that established interactions between exotic plants and soil organisms were disrupted and replaced to some degree by interactions between native plants and soil organisms (Bardgett *et al.* 2013). Native grasses increased at both sites and exotics were reduced leading to shifts in plant inputs, particularly by reducing the proportion of exotic species litter having higher C : N in both sites and increasing the proportion of *S. pulchra* litter with intermediate quality. The reduction of exotic plant inputs followed by replacement by native plant inputs altered the microbial community, increased NO_3_-N availability and nitrification rates and decreased NH_4_-N availability and N mineralization rates. Although no uninvaded grasslands are available as reference sites, these rapid changes indicate that these grasslands have some capacity for soil resilience.

Differences in microbial community biomass, F : B and individual markers (indicated by concentrations of PLFA biomarkers) between unrestored and restored soils support our hypothesis that restoration of the native plant community would decouple the previously existing exotic plant–soil feedbacks and allow for establishment of native plant–soil feedbacks. Potthoff *et al.* (2009) found similar shifts in PLFA profiles that they interpreted to indicate resilience of the soil microbial community to disturbance. Restoration reduced microbial biomass values during germination indicating a stronger response of soil microbes in unrestored soils following the first rains, likely due to higher root activity of germinating exotics, rapid decomposition of exotic annual grassland seedlings due to self-thinning (Bartolome 1979; Savelle 1997; Eviner and Firestone 2007) or decomposition of the previous year's microbial biomass. Shifts in microbial community in response to seasonal changes in temperature and moisture, such as those found here, are expected due to species-specific growth requirements (Pommerville 2007). Mycorrhizal fungi may decline in perennial grassland soils invaded by exotic annual grasses, so we expected fungal biomarkers to be lower in unrestored plots (Hawkes *et al.* 2006). The grasslands of this experiment had reduced fungal biomarkers, 18 : 2 w6c, 18 : 1w9c and 16 : 1 w5cat germination, but increased fungal markers 18 : 2 w6c and 18 : 1w9c the remainder of the season. Similar to Hawkes’ *et al.* (2006) findings, our coastal grassland site had lower AM fungi PLFA markers in unrestored plots dominated by exotic annuals, but this did not occur until late in the growing season (i.e. plant senescence). Native plant species tend to have a later phenology than exotics in the semi-arid grasslands and thus experience peak growth rates later into the season than exotic plants (Jackson and Roy 1986; Holmes and Rice 1996). AM fungi associated with exotics have reduced abundance earlier in the season when their exotic, annual host plants senesce (Nelson and Allen 1993). Other fungal groups were also in high concentration during senescence at both sites. Therefore, phenology of the dominant plant species either annual exotics or native annuals to native perennial grassland species was related to the activity of soil microorganisms.

The anticipated shift in N availability occurred and also showed seasonal patterns corresponding to plant phenologies. Total extractable N and NO_3_-N increased with restoration during peak and senescence periods of the growing season as also observed by Jackson *et al.* (1988, 1989). One of the more striking results was that extractable N concentrations at the inland site were higher in unrestored soils at plant germination but quickly became significantly lower within 2–3 months, suggesting increased rates of N uptake by plants in the unrestored plots. Plants may take up as much $\text{N}{{\text{O}}_{3}}^{-}$ as becomes available (Jackson *et al.* 1988, 1989). This effect of exotic plant removal on increased mineral N has been observed in both grassland and coastal sage scrub in other studies of semi-arid environments (Dickens 2010; Dickens and Allen 2014). In this study, plant uptake of N was still low at germination, but began to increase rapidly as plant growth reached its maximum rates. The peak and senescence sampling dates at these sites correspond to the periods of maximum annual growth and transition to reproduction phases during which their N use would be highest. California grassland natives tend to germinate and complete their life cycles later than exotic annuals (Jackson and Roy 1986; Holmes and Rice 1996) and likely have continued nutrient uptake closer to the senescence sampling date. *Stipa pulchra* recycles about half its annual N internally and thus may not take up N as soon or at rates as high as those observed for exotic annuals (Clark 1977; Jackson *et al.* 1988; Hooper and Vitousek 1998). *Stipa pulchra* is the dominant native so less rapid and total uptake rates would translate to greater overall extractable N left in the soil throughout the season. Restoration led to reduced NH_4_-N and N mineralization but increased nitrification, indicating greater immobilization of N following restoration. In other grasslands, exotic grass invasion is associated with increased N mineralization, which is attributed to a greater abundance of ammonia-oxidizing bacteria (Hawkes *et al.* 2005). Here, the biomass of bacteria was higher in unrestored soils, although our PLFA assay could not identify whether these ammonia-oxidizing bacteria were reduced by restoration.

Long-term invasion and anthropogenic disturbance may be one mechanism explaining the resistance of soil total C and N pools and potential respiration to changes under restored plant community conditions. Total soil C and N pools and C cycling may show resistance or may only be slowly responsive to changes in vegetation. These soils have likely been invaded by exotic annual grasses for more than a century (Minnich 2008), so sufficient time has likely passed for total C and N pools to change in response to invasion. Similar resistance of C and N pools were observed in grasslands of northern California when community composition was altered to test legacy effects of plant–soil interactions (Potthoff *et al.* 2009). However, decreased soil C was found in invaded grasslands in central California compared with reference patches of native grassland (Koteen *et al.* 2011). Kindscher and Tieszen (1998) found that tall grass prairie soils may require >35 years to recover C from agricultural use following restoration. The shifts in litter input at our grasslands may not have been great enough to lead to altered C and N pools. Differences and variability in litter quality in grasslands are often subtle making responses to changes in litter difficult to detect (Eviner and Firestone 2007). This suggests that our grasslands could have been resistant to impacts of the initial exotic annual invasion or that restoration must occur for a longer time than 9 years to detect total soil N and C responses. Another contributing factor is the reinvasion of exotic grass following restoration. While native vegetation continued to dominate, exotic species had a continuous impact on the soil.

Between-site differences in restoration responses of extractable N availability and microbial community structure were primarily seasonal. This corresponded with our hypothesis that soil responses are sensitive to environment. In this case, the important environmental influences included soil nutrient and climatic differences. Greater immobilization at the coastal than the inland site may be the result of a 15 % lower soil C : N ratio and 5 % greater soil organic matter content than at the inland site. Higher organic matter in conjunction with lower C : N soil values would allow for higher rates of N mineralization while also leading to increased immobilization overall in restored plots of the coastal site (Knops *et al.* 2002; Berger and Jackson 2003). Instead, the inland site had a more stable microbial community (F : B was unchanged) and a steady use of nutrients over the season followed by a second peak of microbial activity at senescence. So while differences in soil nutrient conditions, timing of responses to restoration and specific PLFA concentrations occurred between sites, the patterns of increased NO_3_-N and nitrification, reduced N mineralization and altered microbial community composition following restoration occurred at both sites. This indicates that grassland soils were responsive to changes in vegetation and may therefore be resilient to invasion.

## Conclusions

Restoration of invaded grasslands decoupled exotic plant–soil feedbacks related to microbial community structure, extractable N and N cycling. This study indicates that the soils of these systems are dynamic and change in response to exotic or native vegetation type and seasonal variation in soil moisture. Semi-arid grasslands in general are known to be unstable in productivity and reliant on seasonal precipitation patterns (Talbot *et al.* 1939). Measured changes in extractable soil N and microbial characteristics in response to removal of exotic plants indicated that grassland soils are not resistant to the impacts of plant community shifts, but have the capacity for resilience regardless of the method of exotic plant control (i.e. prescribed burn, mowing and weeding). This indicates that the method of exotic plant removal is not important in these grasslands, but that removal of exotic plants and decoupling exotic plant–soil feedbacks are required for grassland soils to diverge from invaded conditions. In contrast, the lack of change in total soil C and N pools and potential soil respiration may be an indication that, for these soil characteristics, these grasslands are resistant to invasion. Stable pools of C and N may buffer these soils, enabling resilience of the more labile and rapidly responding mineral N and microbial characteristics. However, the absence of uninvaded grasslands does not allow us to rule out changes in C and N pools that may have occurred long ago or will require a more complete restoration and longer time frames for recovery. The differences between sites regarding the timing of microbial activity and N cycling highlight the importance of matching sampling efforts to seasonality of plant and microbial growth patterns. Overall NO_3_-N use and net N cycling differences between restored and unrestored plots were similar between the coastal and inland sites, indicating that shifts in plant community composition from exotic to native-dominated communities produce the same impact on N regardless of site history, restoration methods and differences in soil type.

## Sources of Funding

This work has been funded by the University of California-Riverside and specifically the Department of Botany and Plant Sciences and the Center for Conservation Biology, the Shipley-Skinner Riverside County Endowment, the UC Fisher Vegetation Scholarship, The Nature Conservancy and the National Science Foundation (DEB 04-21530).

## Contributions by the Authors

S.J.M.D. and E.B.A. formed the research questions and all authors contributed to the development, analysis of data and manuscript drafting. S.J.M.D. implemented the project in the field and laboratory.

## Conflicts of Interest Statement

None declared.

## Supporting Information

The following Supporting Information is available in the online version of this article –

**Table S1.** The common phospholipid fatty acid (PLFA) biomarkers (μmol PLFA g^−1^ soil) and the corresponding microbial functional groups from the inland site during the 2007–08 season. Asterisks indicate the level of significance between treatments. **P ≤ 0.05.

**Table S2.** The common PLFA biomarkers (μmol g^−1^ soil) and the corresponding microbial functional groups from the coastal site during the 2007–08 season. Asterisks indicate the level of significance between treatments. *P ≤ 0.001, **P ≤ 0.05.

**Table S3.** Soil microbial PLFA principal component (PC) per cent weights at both locations. Positive and negative signs indicate the direction of the weighting along the corresponding PC. Cumulative per cent explained equals the variance within the PLFA data explained by successive PCs.

Additional Information
