# Supplementary material for: Extractable nitrogen and microbial community structure respond to grassland restoration regardless of historical context and soil composition
Source: AoB Plants. 2015 Jan 1;7:plu085. doi: 10.1093/aobpla/plu085 (PMC4323520; doi:10.1093/aobpla/plu085)
Supplement: Additional Information [file supp_7_plu085_index.html]

Extractable nitrogen and microbial community structure respond to grassland restoration regardless of historical context and soil composition — Additional Information 

# Extractable nitrogen and microbial community structure respond to grassland restoration regardless of historical context and soil composition

## Additional Information

Additional Information

**Files in this Data Supplement:**

- Supporting Information - xls file
